# Comparative putative metabolites profiling of *Tachypleus gigas* and *Carcinoscorpius rotundicauda* hemocytes stimulated with lipopolysaccharide

**DOI:** 10.1038/s41598-024-54279-3

**Published:** 2024-02-17

**Authors:** Nurhana Jasni, Chee Lee Wee, Noraznawati Ismail, Nik Soriani Yaacob, Nurulhasanah Othman

**Affiliations:** 1 Institute for Research in Molecular Medicine (INFORMM), Universiti Sains Malaysia, 11800 Gelugor, Malaysia; 2 Department of Chemical Pathology, School of Medical Sciences, Universiti Sains Malaysia, Health Campus, 16150 Kubang Kerian, Malaysia; 3 Institute of Marine Biotechnology, Universiti Malaysia Terengganu, 21030 Kuala Terengganu, Terengganu Malaysia

**Keywords:** Chemical biology, Immunology, Zoology, Molecular medicine

## Abstract

Horseshoe crabs are among the most studied invertebrates due to their unique, innate immune system and biological processes. The metabolomics study was conducted on lipopolysaccharide (LPS)-stimulated and non-stimulated hemocytes isolated from the Malaysian *Tachypleus gigas* and *Carcinoscorpius rotundicauda.* LC–TOF–MS, multivariate analyses, principal component analysis (PCA), and partial least squares-discriminant analysis (PLS-DA) were included in this study to profile the metabolites. A total of 37 metabolites were identified to be differentially abundant and were selected based on VIP > 1. However, of the 37 putative metabolites, only 23 were found to be significant with ANOVA at p < 0.05. The metabolites were identified using several databases, and the literature review of the metabolites was reported in the manuscript. Thus, this study has provided further insights into the putative metabolites' presence in the hemocytes of horseshoe crabs that are stimulated and non-stimulated with LPS and their abundance in each species. Several putative metabolites showed they have medicinal values from previous studies.

## Introduction

Metabolomics is the study of identifying and quantifying small molecules or metabolites in biological systems. Several technologies, such as nuclear magnetic resonance spectroscopy (NMR), gas chromatography-mass spectrometry (GC–MS), and liquid chromatography-mass spectrometry (LC–MS), are used to analyze the metabolites^1,2^. It is essential in clinical research, disease treatment, drug characterization, animal and plant research, agricultural research, and nutrition^1^. Metabolomics can be divided into targeted (analysis of known metabolites) and untargeted metabolomics (analysis of unknown metabolites)^3^. Metabolites are intermediate products of cellular metabolic reactions such as peptides, oligonucleotides, sugars, and amino acids.

Recently, horseshoe crab research has gained popularity among scientists due to its various benefits in the medical field. For example, its blood is widely used as an endotoxin tester in vaccines, drugs, and injectables, as it can clot in the presence of bacteria^4^. Furthermore, many discoveries in human eye research resulted from studies involving nerve pathways of the horseshoe crab eyes^5^. Aside from that, perivitelline fluid (PVF) from a fertilized horseshoe crab egg is rich in vital proteins and amino acids essential for embryogenesis^4^.

Horseshoe crabs belong to the phylum Arthropoda, subphylum Chelicerata, class Merostomata, order Xiphosura, and family Limulidae. Physically, it has a colourless to whitish hemolymph and a brown body consisting of a cephalothorax, abdomen, and swordtail^6^. The global distribution of horseshoe crabs is believed to be constrained by continental geomorphology, temperature barriers, tidal types, and benthic currents^7,8^. *Limulus polyphemus*, *Tachypleus tridentatus*, *T. gigas,* and *C. rotundicauda* are types of horseshoe crab species^9,10^.

Hemocytes are horseshoe crabs' primary regulators of innate immunity^11^. They are susceptible to gram-negative bacterial endotoxins, also known as lipopolysaccharides (LPS), in their outer cell wall. The hemocyte secretes transglutaminase (TGase) and several defence molecules, such as coagulation factors, lectins, antimicrobial peptides, and protein substrates, in response to stimulation by LPS^12,13^. The study of the effect of LPS on hemocytes has been considered one of the most important experimental tools to understand the horseshoe crab immune response. Previous studies by Sarmiento et al.^11^ and Adebayo et al*.*^14^ elucidated hemocytes' transcriptomic and proteomic data stimulated with LPS and non-stimulated hemocytes.

In this study, the metabolomics analysis of two species of horseshoe crab, *T. gigas* and *C. rotundicauda* was conducted. The study was conducted to profile the presence and abundance of the putative metabolites of horseshoe crabs with and without stimulation with LPS and variation among the two species. The study also is looking for metabolites that could have promising potential for medical applications. Analyses were conducted on isolated hemocytes stimulated and non-stimulated with lipopolysaccharides (LPS). LC-TOF–MS and several statistical analyses, such as principal component analysis (PCA), partial least squares discriminant analysis (PLS-DA), ANOVA, Tukey test and t-test, were applied in this study.

## Materials and methods

### Samples preparation, hemocyte culture, and LPS challenge

Three adult horseshoe crabs were collected from the Kuala Kemaman coastal region in Terengganu, Malaysia. Horseshoe crabs were caught during the day and acclimated for 24 h. Each horseshoe crab had 2 mL of hemolymph collected aseptically with pyrogen-free equipment. The hemolymph collection was performed in a biological safety cabinet (ESCO, USA) using patented techniques (MY-155541-A)^11^. The lipopolysaccharides (LPS) challenge on horseshoe crab hemolymph was carried out according to the protocol recently reported by Samiento et al.^11^. Briefly, the hemolymph was mixed with 25 ml 3% NaCl and plated into 6-well culture plates, in triplicates. After 15 min incubation at room temperature, 10^–13^ g/ml of LPS in 2 ml 3% NaCl was added to each well and incubated for 1 h at room temperature. The non-stimulated wells received only 3% NaCl.

### Extraction of metabolite

The extraction was optimized based on Muelas et al.^15^. First, approximately 200 μl of cold extraction solvent [methanol/acetonitrile 50/50 (% v/v)] was added to 600 μl hemocytes stimulated and non-stimulated samples. The samples were mixed vigorously by vortex for approximately 30 s. After vigorously mixed, the samples were incubated at − 20 °C for 20 min. The samples were centrifuged at 13,000 rpm for 5 min at 4 °C. After being centrifuged, the supernatant was transferred and dried using a speed vacuum. The methanol extracts were stored at − 80 °C until the sample analysis.

### Analysis of LC-TOF–MS

The mass spectrometry analysis was performed at the Institute of Systems Biology (INBIOSIS), Universiti Kebangsaan Malaysia. Three biological and six technical replicates from each group were analyzed using LC-TOF–MS. The analyses were performed using ultra-high-performance liquid chromatography (UHPLC) with a microTOF Q III mass spectrometer (MS) (Bruker Daltonics, Bremen, Germany) equipped with an electrospray source (ESI) and connected to an Ultimate 3000 UHPLC system (Dionex, Sunnyvale, CA, USA) equipped with an Acclaims Polar Advantage II, 3 × 150 mm, 3-μl particle size C18, reverse-phase column at the flow rate of 0.4 ml/min at 40 °C with a sample injection volume of 3 μl. The running buffers were (A) deionized distilled water with 0.1% formic acid and (B) 100% acetonitrile with a 22-min total run time. The gradient elution was carried out from 5 to 80% buffer B over 22 min as follows: 5% B (0–3 min), 80% B (3–10 min), 80% B (10–15 min), 5% B (15–22 min). MS was performed in ESI positive ionization mode with the capillary voltage at 4,500 V, nebulizer pressure at 1.2 bar, and drying gas at 8 L/min in 200 °C. The scan range was from 100 to 1000 *m/z*. Data processing was performed using the Data Analysis 4.0 and Profile Analysis (Bruker Daltonics) software. Lastly, the graphical representation was performed on WEGO for data analysis of the sample. The caffeic acid standard was used as a control to ensure data quality by providing the optimum condition of extraction and instrument.

### Statistical analysis

Multivariate statistical evaluation of the preprocessed metabolic profiling data was performed with SIMCA-P + (version 12) (Umetrics, Umea, Sweden). Bucketing was generated using Data Analysis 4.0 and Profile Analysis (Bruker Daltonics). The differential metabolites obtained from the multivariate data analysis software were validated using ANOVA with post hoc Tukey's tests and t-tests. All metabolites contributing to group separation were significant at p < 0.05. The Permutation test was performed for validation.

### Compound identification

Compound identification of metabolites was performed by comparing the accuracy of the *m/z* value < 10 ppm and MS/MS spectra with available online databases: Human Metabolome Database (HMDB)^16^, Metabolite and Chemical Entity Database (METLIN)^17^, KEGG^18^ and LIPIDMAPS^19^ and MassBank. KEGG pathway analysis was used to determine the putative metabolite pathway.

### Pathway enrichment analysis

Pathway enrichment analysis was performed by analyzing data on putative metabolites, which were significant with ANOVA using MetaboAnalyst version 5.0.

## Results and discussion

### Chromatogram and multivariate analysis

Two forms of the horseshoe crab hemocyte metabolites were reported: the non-stimulated and stimulated with LPS. LPS is an essential outer membrane component of gram-negative bacteria, which consists of Lipid A, O-antigen, and hydrophilic core polysaccharide^20^. It is a primary factor in hemocyte activation and is regarded as one of the most important experimental tools for understanding the horseshoe crab immune response.

**Figure 1:**
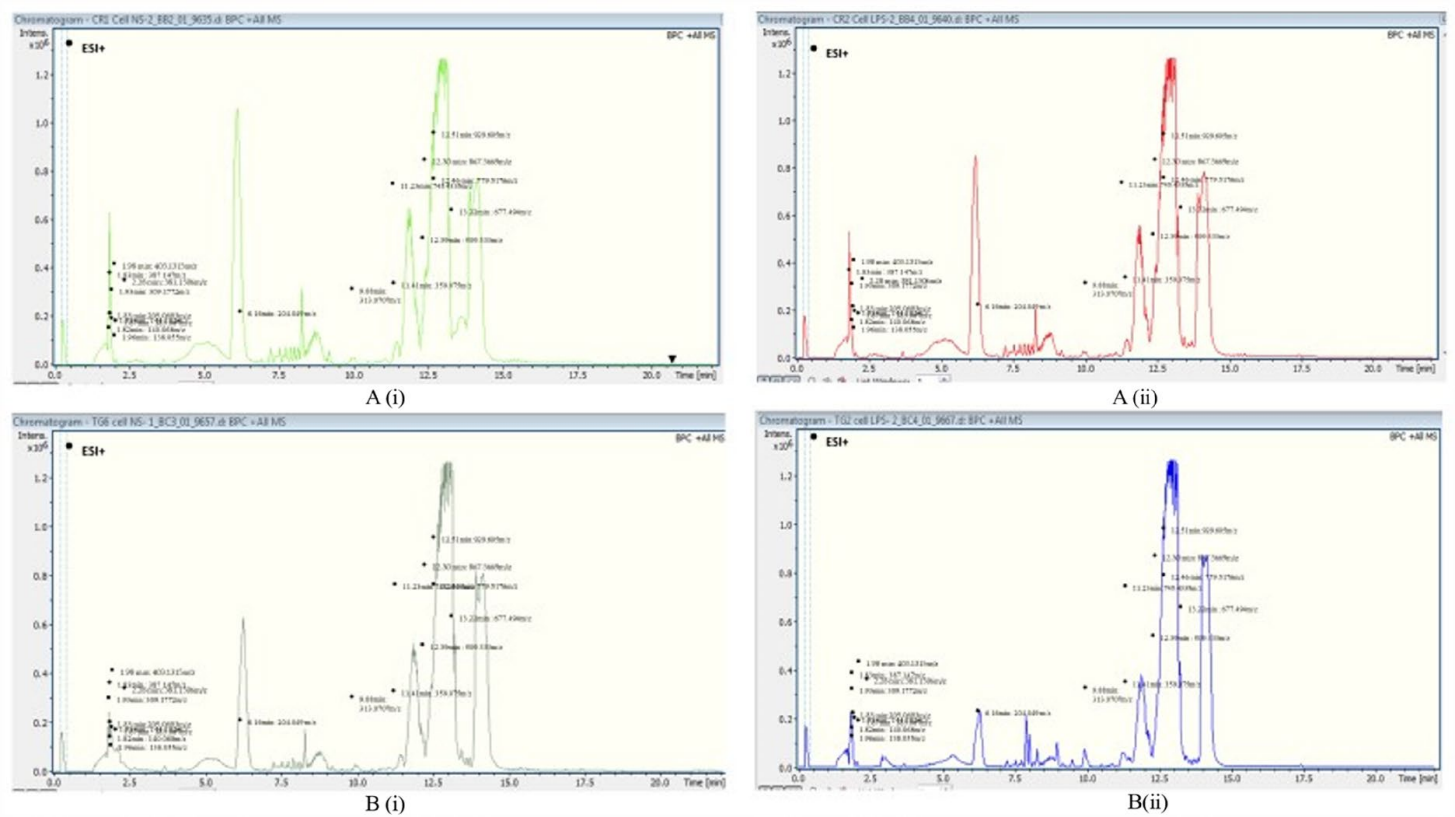
The base peak chromatogram (BPC) of non-stimulated hemocyte cells of *C. rotundicauda* **A(i)** vs hemocytes stimulated with LPS **A(ii)** and non -stimulated hemocyte cells of *T. gigas* **B(i)** vs stimulated with LPS **B(ii)** by Liquid Chromatography-Time of Flight-Mass Spectrometry (LC–TOF–MS).

The metabolomics studies used a high throughput method (LC–TOF–MS) and multivariate analyses (PCA and PLS-DA). Based on the differences in the peak and area of the chromatogram observed following LC–TOF–MS analysis in Fig. 1, there are differences in the putative metabolite profile in both species stimulated and non-stimulated hemocytes. Multivariate analyses were then conducted to get a general overview and understanding of the spread of variability in the data.

This study used PCA and PLS-DA to analyze the preprocessed LC–TOF–MS datasets. The PCA model reveals the general metabolic information and visually eliminates abnormal sample data. It was also conducted to determine the global differences between the metabolic profiles of the groups. Based on the analyses, all samples from the two species appeared in the Hotelling T2 with 95% confidence, suggesting that all the samples can be used for further research. The parameters described the PCA model (R^2^X = 0.0918, Q^2^ = 0.246).

**Figure 2:**
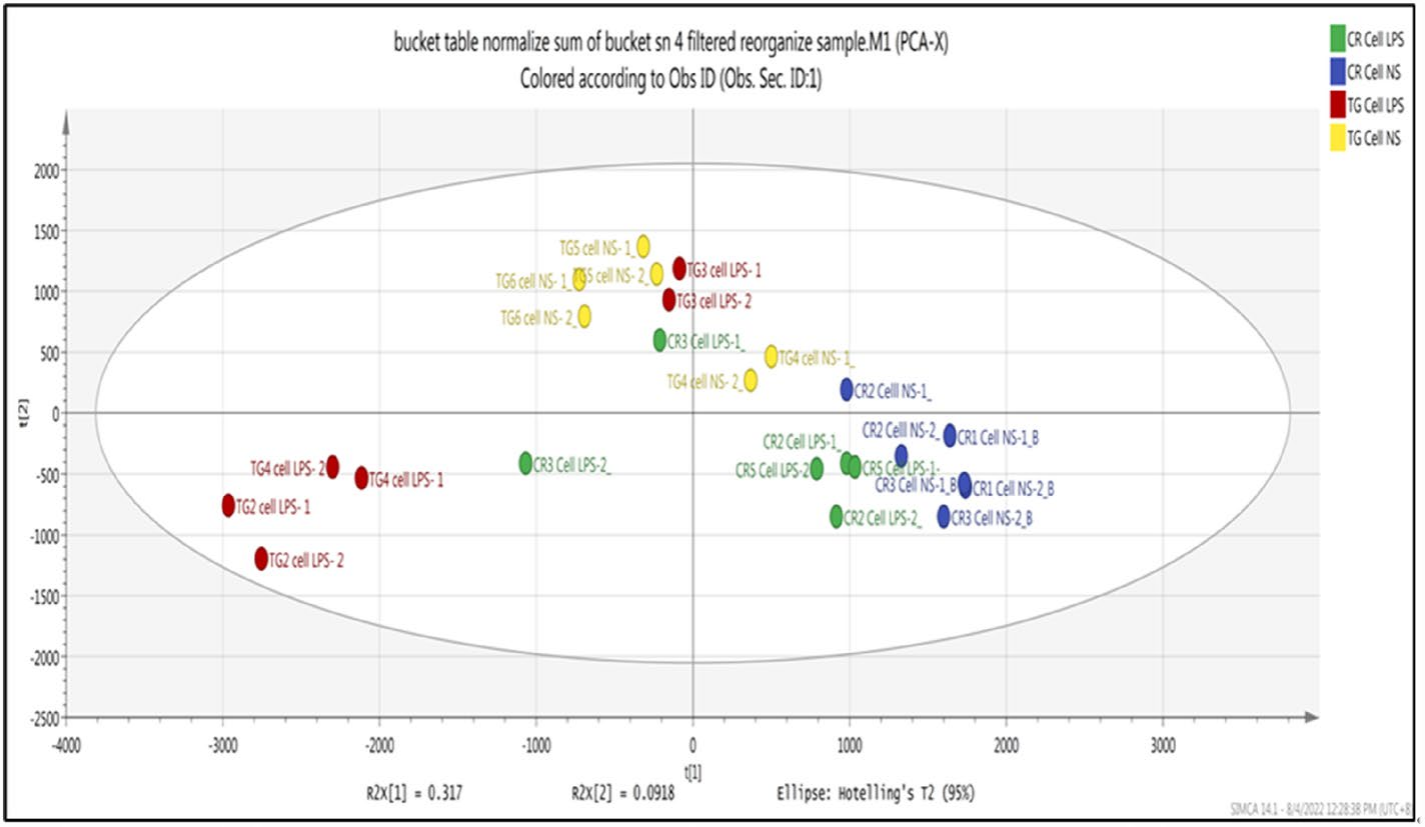
PCA score plot of PC1 versus PC2 scores for compounds or metabolites detected in each treatment: Hemocytes of *C. rotundicauda* and *T.gigas* non-stimulated and stimulated with LPS.

As depicted in Fig. 2, the PCA plot shows four different groups of the samples, which are *C. rotundicauda* stimulated with LPS (green), non-stimulated *C. rotundicauda* (blue), *T. gigas* stimulated with LPS (red) and non -stimulated *T. gigas (*yellow). Based on the plot, there are differences between both horseshoe crab species, which can be measured from the locations of the metabolite's distributions in the quadrant. The *C. rotundicauda* (green and blue) samples were clustered on the lower quadrant on the right side, while the *T. gigas* (yellow and red) samples were clustered on the left upper and lower quadrants.

Comparison between LPS-stimulated (red) and non-stimulated hemocytes (yellow) of *T. gigas,* reveals an apparent difference in the metabolites produced. In contrast, in *C. rotundicauda*, there is no evident difference in the metabolites produced by the hemocytes stimulated with LPS (green) and without stimulation with LPS (blue), as they were grouped within the same quadrant.

**Figure 3:**
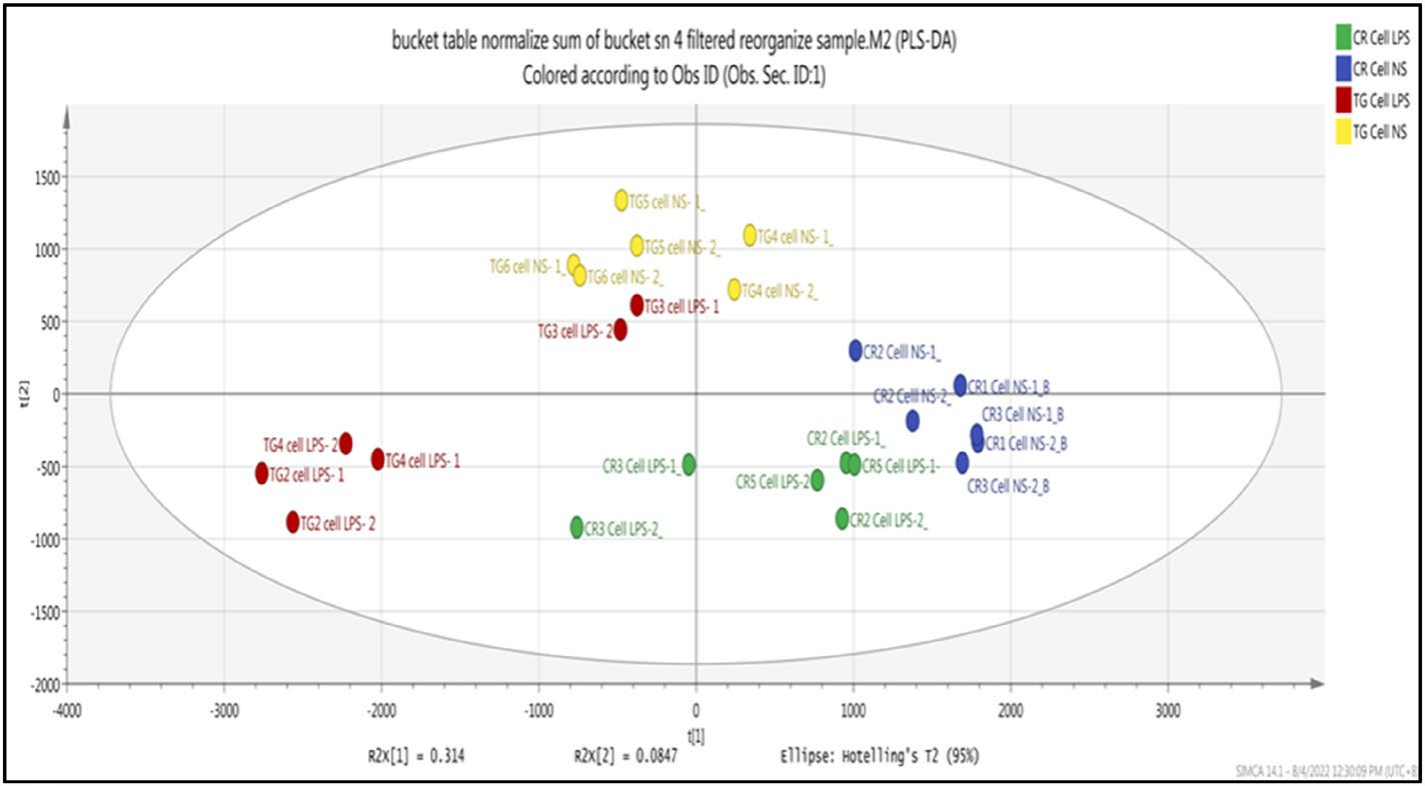
PLS-DA score plots for compounds or metabolites detected in each treatment*:* Hemocytes of *C. rotundicauda* and *T. gigas* non-stimulated and stimulated with LPS (*C. rotundicauda* stimulated with LPS (green*), C. rotundicauda* non-stimulated (blue), *T. gigas* stimulated with LPS (red), and *T. gigas* non-stimulated (yellow).

The PLS-DA model analysis further demonstrated distinct discrimination in the metabolomic changes between the two species (Fig. 3). The acceptable values for the intercepts R^2^ (cum), the goodness of fit, was 0.99, and Q^2^ (cum), predictability, was 0.78. Model cross-validation through permutation tests (100 permutations) and sevenfold cross-validation generated the intercept R^2^ and Q^2^ 0.978 and 0.267, respectively. The results show that the PLS-DA model is not overfitting and is valid for this metabolomic profiling.

The data presented herein demonstrated a clear and significant separation between the two species of horseshoe crabs and significant differences in the putative metabolites of the stimulated and non-stimulated hemocytes of *T. gigas* by multivariate analyses using PCA and PLS-DA.

**Figure 4:**
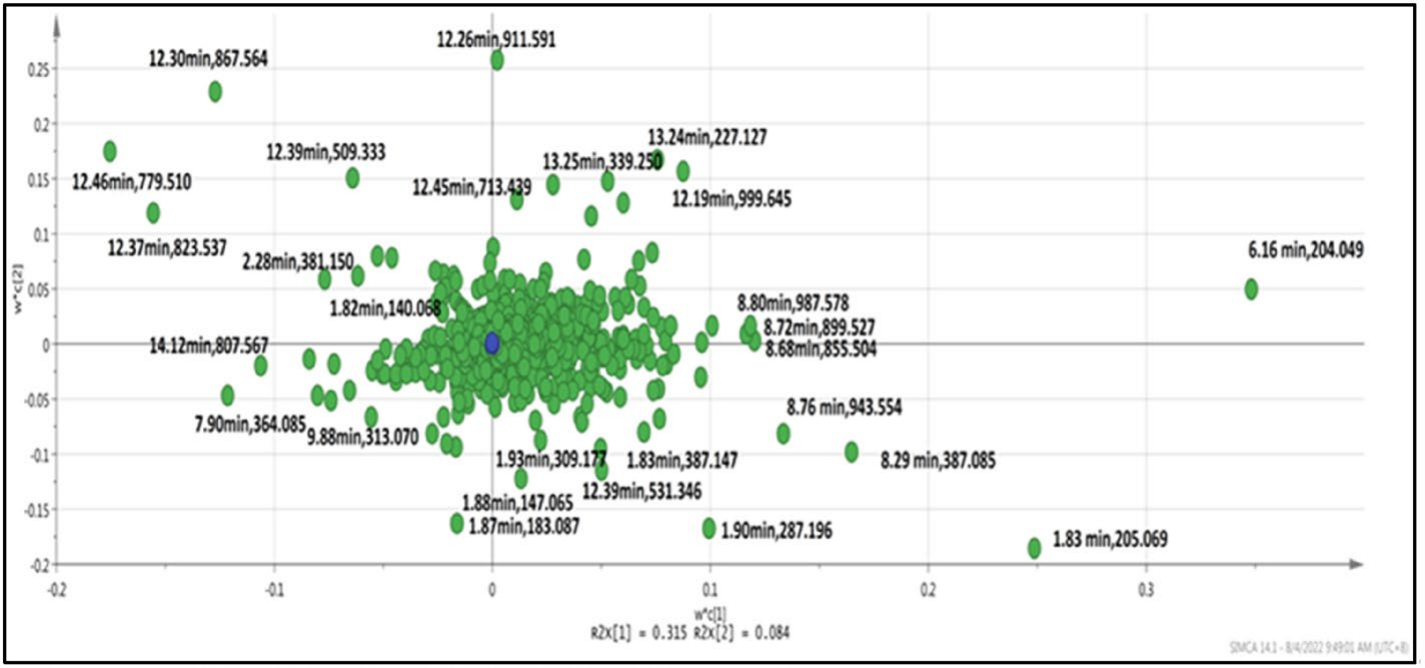
Loading plots of PLS-DA for metabolites (masses) detected via untargeted LC–TOF–MS in different treatments. The green dots labelled with retention time represent the masses distributed w*c(1) and w*c(2) planes and the highest VIP (Variables Importance for the Projection) > 1. Blue dots represent the origin.

PLS-DA also allows for determining and discriminating metabolites using the variable importance on projection, known as VIP. The VIP score value indicates the contribution of a variable to the discrimination between all the classes of samples. Mathematically, these scores are calculated for each variable as a weighted sum of squares of PLS weights. The mean VIP value is 1, and VIP values over one are usually considered significant. A high score agrees with a solid discriminatory ability and thus constitutes a criterion for selecting biomarkers. The discriminating metabolites were obtained using a statistically significant threshold of VIP values obtained from the PLS-DA model on the normalized raw data at the univariate analysis level. The P value was calculated by one-way analysis of variance (ANOVA) for four groups analysis. Metabolites with VIP values greater than 1.0 and p-values less than 0.05 were considered statistically significant metabolites. Figure 4 depicts a PLS-DA loading plot showing the variables that contributed to separating the four groups.

Based on Fig. 4, the farther the point is from the origin, the greater the weight value or the greater the effect of determining the grouping of the samples. In the non-stimulated *C. rotundicauda* hemocytes*,* the metabolite masses that distinguished it from the other three groups are 6.16 min:204.0492 *m/z*, 1.83 min:205.0693 *m/z*, 8.29 min:387.0849 *m/z*, 8.76 min:943.5543 *m/z*, 1.83 min: 387.1471 *m/z*. On the other hand, these metabolite masses; 1.90 min: 287.1969 *m/z*; 1.88 min: 147.0650 *m/z*; 12.39 min: 531.3464 *m/z*; 1.93 min: 309.1772 *m/z*; were unique, and highly abundant in LPS stimulated *C. rotundicauda* hemocytes. For the group of non-stimulated hemocytes of *T. gigas*, the metabolites that contributed to the separation were as follows: 12.26 min:911.5912; 13.24 min: 227.1270; 13.25 min: 339.2501 *m/z*; 12.45 min:713.4391 *m/z*; 12.19 min:999.6454 *m/z* and 12.39 min: 509.3332 *m/z*. Lastly, the group of metabolites from LPS-stimulated *T. gigas* hemocytes was separated from the other groups due to the following metabolite masses: 12.37 min:823.5371 *m/z*; 12.46 min: 779.510 *m/z*; 7.90 min: 364.0847 *m/z*; 14.12 min:807.5674 *m/z*; 11.41 min: 359.0756 *m/z*; 9.88 min: 313.0707 *m/z*; 2.45 min: 345.0350 *m/z*. All details can be found in Table 1.

**Table 1:** Putatively identified metabolites with the highest variable importance on projection (VIP) score as determined by a partial least square-discriminant analysis (PLS-DA) and one-way ANOVA.

No	The retention time of the precursor ion and mass charge ratio (*m/z*)	Vip score	Putatively identified metabolites	MS/MS formation of products ions	Adducts (*m/z*)	ANOVA	
						(P value < 0.05)	Significance
1	6.16 min: 204.0492	7.28253	7,8-Dihydroxanthopterin	103.0544 680 104.0613 2535 123.0601 501 167.0148 1016 187.0221 35,137 188.0255 3296 189.0208 679 204.0487 3207 205.0422 516 228.0484 4066 120.0572 7106	[M + Na]^+^ 204.0492	< 0.0001	Yes
2	2.28 min: 381.1506	6.40998	(N-(1-Deoxy-1-fructosyl) tryptophan)	114.0590 6418 116.1064 2513 132.0655 21,887 134.1171 2386 160.0609 42,542 161.0640 2592 203.1746 3056 291.1540 8916 335.1459 3214 381.1451 1630	[M + H^+^]^+^ 381.1506	0.2812	No
3	1.83 min: 205.0693	6.40523	Harman	144.0925 102 187.0224 354 205.0684 704 104.0614 266 105.0654 266 168.0185 301 169.0100 283 186.0293 2819 187.0237 2868 203.0573 189	[M + Na]^+^ 205.0693	< 0.0001	Yes
4	1.98 min: 403.1315	5.86116	4-Hydroxy-5-(3'',5''-dihydroxyphenyl)-valeric acid-O-glucuronide	244.0827 308 301.1382 341 313.1453 323 345.1268 2780 346.1325 410 359.1393 357 403.132312348 404.1363 1612 405.2604 3342 406.2692 301	[M + H^+^]^+^ 403.1315	0.5227	No
5	12.30 min: 867.5669	5.65392	Phosphatidylcholine, PC(DiMe(11,5)/DiMe (9,3))	121.0650 3741 131.0700 8012 131.0700 8012 133.0850 57,790 147.0794 8196 165.0892 11,001 175.0967 8458 177.1110 24,465 233.1875 31,999 259.2029 7867 277.2136 56,231 278.2165 9110	[M + H^+^]^+^ 867.5669	0.0347	Yes
6	12.26 min: 911.5912	5.44306	Phosphatidylinositol PI (18:3(6Z,9Z,12Z)/22:3(10Z,13Z,16Z))	131.0718 22,539 133.0875 169,846 147.0815 19,251 165.0926 26,868 175.0992 24,929 177.1140 72,284 233.1917 89,021 259.2065 19,939 277.2182 153,517 278.2216 26,218	[M + H^+^]^+^ 911.5912	0.0036	Yes
7	12.39 min: 509.3332	5.44194	Contignasterol	121.0600 611 133.0799 141 165.0847 195 204.0420 214 222.0531 644 240.0626 212 506.3170 336 507.3174 295 508.3171 129	[M + H^+^]^+^ 509.3332	0.1314	No
8	12.46 min: 779.5176	4.9829	Phosphatidylglycerol PG (15:1(9Z) \/22:6(4Z,7Z,10Z,13Z,16Z19Z))	121.0661 11,293 131.0716 7804 133.0876 75,318 147.0821 10,764 165.0927 15,336 177.1142 28,197 233.1914 49,959 259.2065 7864 277.2181 77,415 278.2204 13,709	[M + H^+^]^+^ 779.5176	< 0.0001	Yes
9	1.90 min: 287.1969	4.84398	Androstenedione	91.2931 54 144.1013 35,752 145.1053 2676 146.1053 219 158.1162 63 127.0366 120 129.0443 126 184.1025 125 198.1078 421 229.0910 80 230.1004 1574 231.1098 176	[M + H^+^]^+^ 287.1969	0.0085	Yes
10	8.29 min: 387.0849	4.5821	Methyl 18-bromo-15E,17E-octadecadien-5,7-diynoate	139.0201 363 155.0151 178 167.0174 160 193.1047 172 386.1375 114 139.0208 21,833 155.0158 6077 166.0317 3802 167.0173 7813 193.1006 4877 196.0427 2732 212.0358 1768 286.0895 2645 350.0507 1899 387.0825 5835	[M + Na]^+^ 387.0849	0.0029	Yes
11	12.19 min: 999.6454	4.5019	Phosphatadylinositol PI (22:0\/22:1(11Z))	130.1583 2132 131.0708 437 131.1613 294 133.0854 2381 134.0836 501 175.0944 611 177.1124 1330 219.1254 185 221.1368 269 303.2310 152 259.2045 109 138.0573 129 286.0895 2645 350.0507 1899 387.0825 5835	[M + Na]^+^ 387.0849	0.0515	No
12	12.37 min: 823.5371	4.35623	Phosphatadylinositol PI(P-18:0V16:0)	130.1583 2132 131.0708 437 131.1613 294 121.0643 63,981 131.0698 62,417 133.0857 515,436 134.0895 6322 147.0797 71,183 165.0901 100,118 175.0971 16,567 177.1115 208,522 233.1884 326,061 259.2035 62,890 277.2146 509,259 278.2175 89,508 178.1137 3233 219.1219 4533 221.1379 6559 303.2291 3347	[M + H^+^]^+^ 823.5371	< 0.0001	Yes
13	1.93 min: 144.1021	4.32857	Proline betaine (stachydrine)	116.0969 86 128.0700 91 143.4479 80 144.1021 120,466	[M + H^+^]^+^ 144.1021	0.1209	No
14	13.24 min: 227.1267	4.25118	3,4-Methylenesebacic acid	103.0541 1402 119.0597 1793 121.0656 188,162 122.0682 14,759 123.0699 501 133.0853 1401 139.0752 814 147.0803 3312 165.0912 10,779 166.0937 994	[M + H^+^]^+^ 227.1267	0.0307	Yes
15	13.22 min: 677.4942	4.17901	Diglyceride DG (18:2n6/0:0/20:4n6)	121.0652 1062 133.0863 2207 165.0923 2022 177.1133 704 227.1291 3950 291.2317 992 321.3149 778 338.3432 2519 679.4634 4530 680.4670 1240	[M + Na]^+^ 677.4942	0.0204	Yes
16	12.39 min: 531.3464	4.16397	Nerolidol-3-O-α-L-rhamnopyranosyl-(1 → 6)-β-D-glucopyranoside	121.0656 609 133.0854 1273 153.5986 214 165.0907 392 177.1114 620 221.1379 167 233.1931 465 277.2150 590 407.2084 170 451.2294 213	[M + H^+^]^+^ 531.3464	0.3543	No
17	12.22 min: 955.6180	4.14473	Phospholipid inositol PI (21:0\/20:2(11Z,14Z))	130.1614 932 131.0719 378 133.0866 1326 134.0867 175 138.0632 102 175.0985 191 177.1121 631 178.1205 96 219.1314 145 221.1462 99 147.0815 31,073 165.0930 42,067 233.1916 146,259 259.2064 34,867 277.2181 264,907 278.2213 44,941	[M + Na]^+^ 955.6180	0.0377	Yes
18	12.55 min: 885.5786	4.09083	Phospholipid inositol PI (22:4(7Z,10Z,13Z,16Z) \/16:1(9Z))	133.0832 164 386.2859 128 412.3203 155 133.0877 147 177.1085 116 506.3201 164 131.0689 179 133.0853 683 175.1042 110 177.1093 328	[M + H^+^]^+^ 885.5786	0.1305	No
19	7.90 min: 364.0847	4.05784	4-Methylthiobutyl-desulfoglucosinolate	130.1614 932 131.0719 378 133.0866 1326 134.0867 175 107.0493 10,733 118.0649 2314 121.0884 5092 153.0790 2706 155.0169 30,040 167.0166 2286 182.0278 5312 253.0877 2140 265.0861 4191 346.0747 7704	[M + Na]^+^ 364.0847	0.0191	Yes
20	1.83 min: 387.1471	3.68869	N-acetylactosamine	140.0654 116 200.9894 216 202.0630 566 202.5587 125 205.0685 55,059 206.0710 3773 207.0723 724 246.0931 535 265.0935 149 385.3360 115 160.0602 299 161.0623 541 132.0675 454 133.0699 200 202.0601 15,537 202.5606 2426 203.0618 674 204.0568 302 205.0685 80,445 206.0713 4698 207.0699 898 222.5715 1649 246.0947 835	[M + H^+^]^+^ 387.1471	0.0254	Yes
21	13.25 min: 339.2501	3.59053	5,6-DHET	121.0652 2137 122.0671 224 124.0814 316 133.0847 873 134.0900 123 153.5960 182 165.0915 402 166.6076 146 177.1097 392 283.1752 108	[M + H^+^]^+^ 339.2501	0.0009	Yes
22	1.87 min: 183.0870	3.51368	L-Iditol	111.0450 363 117.0520 212 129.0535 747 147.0656 361 165.0734 150 137.1095 151 138.1307 168		0.0270	Yes
23	12.45 min: 713.4391	3.45403	PG (18:4(6Z,9Z,12Z,15Z}) \/14:1(9Z))	112.1125 767 113.1097 122 114.1268 2824 115.1332 132 133.0888 103 156.1376 125 277.2215 140 713.4391 1083 714.4375 417 715.4301 94	[M + H^+^]^+^ 713.4391	0.4048	No
24	2.33 min: 293.1001	3.33454	Canavaninosuccinate	114.0545 1053 132.0650 12,209 133.0684 927 134.0489 140 160.0606 17,940 161.0619 942 247.0908 414 293.1027 291 294.1463 133	[M + H^+^]^+^ 293.1001	0.2252	No
25	8.76 min: 943.5543	3.27538	Glycerophosphoinositolphosphate PIP (16:0/20:2(11Z,14Z))	158.9631 1585 226.9487 5942 227.9509 257 294.9426 321 362.9223 1983 363.9323 155 430.9121 2303 431.9123 170 498.8967 744 566.8818 427	[M + H^+^]^+^ 943.5543	0.0003	Yes
26	11.52 min: 437.2495	3.04065	Stearoylglycerone phosphate	133.0896 62 155.0076 74 173.0924 114 191.1009 67 263.2400 79 275.2244 75 337.2766 105 338.2671 66 437.2468 1202 438.2495 310	[M + H^+^]^+^ 437.2495	0.2637	No
27	12.51 min: 929.6049	2.92174	Glycerophosphoinositol PI (19:0\/22:4 (7Z,10Z,13Z,16Z))	No fragment	[M + H^+^]^+^ 929.6049	0.7124	No
28	11.23 min: 745.4335	2.84363	Octaprenyl diphosphate	358.0153 118 102.0664 1215 124.0798 4535 124.5817 591 133.0845 7583 144.0750 556 144.5918 1263 146.0924 1996 166.6043 698 168.1039 621 177.1098 2199	[M + Na]^+^ 745.4335	0.0007	Yes
29	13.38 min: 373.2712	2.78079	Cervonoyl ethanolamide	145.1025 4905 159.1184 8316 213.1647 9697 245.1556 6860 247.1697 5917 261.1866 6015 313.2182 5560 337.2533 8287 355.2652 69,327 356.2690 16,173	M + H^+^]^+^ 373.2712	0.1507	No
30	1.88 min: 147.0650	2.64587	Mevaldate	129.0520 461 140.5378 108 144.1009 78,594 145.1037 5918 146.1034 544 147.0646 1136 148.0868 212 152.0467 169 172.5595 298	[M + H^+^]^+^ 147.0650	0.0287	Yes
31	1.82 min: 140.0685	2.41111	Valine	116.0708 432 135.0461 118 138.0576 101 139.1227 1882 140.1368 347	[M + Na]^+^ 140.0685	0.0151	Yes
32	1.93 min: 309.1772	2.33719	Fructoselysine	305.1656 233 307.1545 1682 308.1551 166 184.0919 131 122.0824 1028 123.0863 149 144.1058 504 162.1147 129 166.0885 151 185.0310 271 202.0566 167 305.1537 184 132.1120 113 130.0534 123 134.0480 298 177.0362 100 179.0511 106 178.9559 106 252.9233 114 240.0995 185	[M + H^+^]^+^ 309.1772	0.0431	Yes
33	1.96 min: 138.0558	2.31838	Anthranilate	106.0268 165 124.0357 147 138.0540 36,259	[M + H^+^]^+^ 138.0558	0.0051	Yes
34	14.12 min: 807.5674	2.28644	PA (22:1(11Z) \/22:4(7Z,10Z,13Z,16Z))-Diacylglycerophospholipid	415.2818 350,041 416.2845 78,045 417.2869 9342 418.2901 890 433.2920 78,504 434.2942 18,130 435.2953 2345 456.3075 48,422 457.3099 12,471 458.3134 176 357.2784 137,388 358.2812 33,427 375.2874 9084 767.5770 9551 785.5898 543,235 786.5930 285,219 787.5955 77,674 788.5969 1142	[M + H^+^]^+^ 807.5674	< 0.0001	No
35	11.41 min: 359.0756	2.05207	7,8-Dihydroneopterin 2'',3''-cyclic phosphate	133.0298 2519 161.0235 2642 163.0390 15,913 179.0341 4141 181.0489 2180 187.0391 7848 191.0337 2077 295.0606 3710 323.0549 4970 341.0663 2201 299.1840 2416	[M + ACN + H^+^]^+^ 359.0756	0.0144	Yes
36	9.88 min: 313.0707	2.00141	(Indole-3-acetyl) aspartic acid	160.0533 2538 205.0664 1927 223.0756 8925 233.0599 3569 249.0547 2000 251.0709 16,942 252.0746 2638 269.0816 4896 277.0512 2309 295.0597 7017	[M + Na]^+^ 313.0707	0.1294	No
37	2.45 min: 345.0350	1.71813	dtMP Deoxythymidylic acid	98.9856 209 173.0214 3249 174.0278 12	[M + Na]^+^ 345.0350	0.0010	Yes

### Compound identification

Identification of the compounds was performed by comparing the accuracy of the *m/z* value (< 10 ppm) and MS/MS spectra with available online databases: Human Metabolome Database (HMDB)^16^, Metabolite and Chemical Entity Database (METLIN)^17^, KEGG^18^, LIPIDMAPS^19^ and MassBank. All the putatively identified metabolites are reported in Table 1.

### Comparative metabolomics analyses

An untargeted metabolite profiling of two horseshoe crab species, *C. rotundicauda,* and *T. gigas*, was performed in this study. There were two conditions observed: stimulated and non-stimulated with LPS. Multivariate statistical analyses such as PCA, PLS-DA, ANOVA, Post Hoc Tukey test and t-test were conducted. Based on the PLS-DA analysis, 37 metabolites are in the VIP > 1 category. Of 37 metabolites, only 23 were statistically significant when ANOVA was performed at p-value < 0.05. All the putative metabolites were tabulated in Table 1. Further statistical analysis using Post Hoc Tukey was also conducted after performing ANOVA.

**Figure 5:**
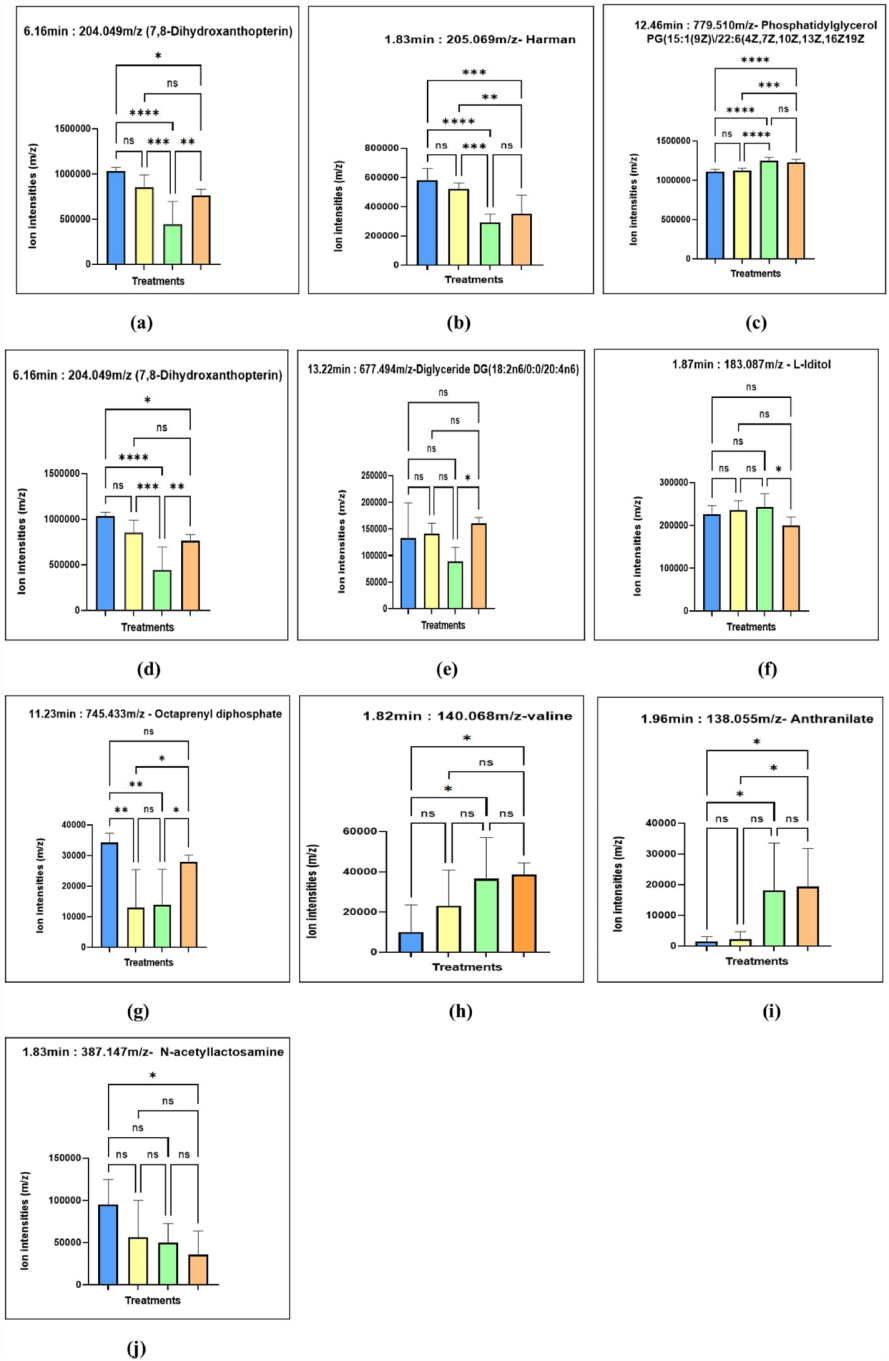
Figure shows the representative ion intensities for *m/z* value (**a**) 7,8-Dihydroxanthopterin (287.196 *m/z*), (**b**) Harman (205.0693 *m/z*), (**c**) Phosphatidylglycerol PG (15:1(9Z)\/22:6(4Z,7Z,10Z,13Z,16Z19Z)) (779.5176 *m/z*), (**d**) Androstenedione (287.1969 *m/z*), (**e**) Diglyceride DG(18:2n6/0:0/20:4n6) (677.4942 *m/z*), (**f**) L-Iditol (183.0870 *m/z*), (**g**) Octaprenyl diphosphate (373.2712 *m/z*), (**h**) Valine (140.0685 *m/z*), (**i**) Anthranilate (138.0558 *m/z*), (**j**) N-acetyllactosamine (387.147 *m/z*). The blue colour indicates *C. rotundicauda* non-stimulated, yellow for *C-rotundicauda* stimulated, green for *T. gigas* non-stimulated, and orange for *T. gigas* stimulated.

Of 23, 7, 8-Dihydroxanthopterin, Fig. 5a is one of the putative metabolites found to be significant in both tests. No research studies have reported on this metabolite in horseshoe crabs. However, several studies on this metabolite from other organisms have been found. For example, in *Stizostedion lucioperca,* this metabolite influences the eye colour and vision of the fish^21^. The guanine crystals form a reflective layer that produces the silvery colour present on the eye surface. Meanwhile, the block-shaped crystals backscatter light into the retina, which helps to increase the sensitivity to light.

In human studies, this putative metabolite was found in the urine of phenylketonuria and lethal hyperphenylalaninemia patients^22^. It was reported to play a role in the pathogenesis of neurological symptoms in both diseases. Figure 5a shows that this putative metabolite was reduced in both species' hemocytes after LPS stimulation. However, Tukey's multiple comparisons test shows that the reduction of metabolites after stimulation with LPS is not statistically significant in *C. rotundicauda* but statistically significant in *T. gigas.* A comparison between species using Tukey's also shows significant differences between both species, and this metabolite can be found more abundantly in *C. rotundicauda* than in *T. gigas.*

Next, Harman, Fig. 5b, a natural B-carboline alkaloid becoming interesting due to its anti-cancer properties^23^. It also was found to be decreased in *C. rotundicauda* and *T. gigas* after hemocytes were stimulated with LPS. However, despite reductions, Tukey's multiple comparisons test shows the reduction is insignificant in each species between stimulated and non-stimulated forms. Despite that, there are significant differences in the metabolite between both species in which this metabolite is abundant in *C. rotundicauda.* By knowing this, isolation of the metabolites can be suggested on *C. rotundicauda* instead of *T. gigas*. This metabolite is usually known to be derived from plants, and sesame seed oil was reported to have high levels of β-carbolines^24^. The fungi entomopathogen *Conidiobolus coronatus*^25^ can also secrete it. It is also considered a nonpolar heterocyclic aromatic amine with potential mutagenicity^24^. It is also a reversible competitive monoamine oxidase inhibitor, increasing serum serotonin concentrations in tissues^25^. Antibacterial activities of Harman analogues against four gram-positive and two gram-negative bacteria damaged bacterial cell membranes and walls and disrupted the function of type II topoisomerase^26^. These derivatives also have potential as new bactericides and antibiotics, as the in-vivo antibacterial assay shows a protective efficacy of 81%^26^. In insects, Harman also resulted in delayed pupation and adult eclosion and inhibited total monoamine oxidase activity^25^.

Phosphatidylglycerol, Fig. 5c, increased in hemocytes after stimulation with LPS in both species. However, despite having an increment, Tukey's multiple comparisons test shows the increment is insignificant in both species stimulated and non-stimulated groups. Despite that, there is a significant difference if comparing two species in which this metabolite can be found to be abundant in *T. gigas.* Phosphatidylglycerol (PG) is a naturally occurring phospholipid and is essential for the growth and photosynthesis of photosynthetic organisms^27^. It is the only major phospholipid in the thylakoid membrane of chloroplasts^27,28^. As it is crucial for photosynthesis, the loss of PG in *Arabidopsis thaliana* resulted in severe defects in the growth and development of chloroplast with decreased accumulation of chlorophyll, impaired thylakoid formation, and also downregulation of photosynthesis-associated genes encoded in nuclear and plastid genomes^28^. PG is also one of the components needed in daptomycin to exert its antibacterial effect^29^. PG and sulfoquinovosyldiacylglycerol (SQDG) have similar physicochemical properties, bilayer thickness, and bending rigidity^30^. However, the function of this metabolite in horseshoe crabs should be further elucidated.

Androstenedione, Fig. 5d increased after the stimulation of hemocytes with LPS in both species. However, the Tukey test shows that this increment is not statistically significant. Despite that, both species have a significant difference in metabolites. In *C. rotundicauda* hemocyte, this metabolite is abundant compared to in *T. gigas.* Thus, if Androstenedione isolation is yet to be performed, it can be isolated from *C. rotundicauda.* Several studies have reported on androstenedione, but no specific analysis of androstenedione of horseshoe crabs has previously been reported. Dong-Ma et al. reported that androstenedione and androgens androstenedione (ADD) are predominant steroid hormones in surface water or wastewater and can disrupt the endocrine system in fish^31^. Androstenedione is produced in male and female gonads and the adrenal glands and is known for its crucial role in producing estrogen and testosterone^32^. It is also a precursor for several steroid substances like testosterone, estradiol, ethinyl estradiol, testolactone, progesterone, cortisone, cortisol, prednisone, and prednisolone^33^. It is also sold as an oral supplement to increase testosterone levels^32^. The supplement can also lower triglycerides (TG) and high-density lipoprotein (HDL) cholesterol, increase oestradiol concentration, and is a natural alternative to an anabolic steroid^32,34^. Other uses of the metabolite include as an enhancer for athletic performance, building body muscles, reducing fats, increasing energy, maintaining healthy RBCs, and increasing sexual performance^32^. Androstenedione is also listed among performance-enhancing drugs (PEDs). However, it was banned by the World Anti-Doping Agency and International Olympic Committee^32^.

Diglycerides, Fig. 5e, were found to be increased after stimulation with LPS in *C. rotundicauda;* meanwhile, in *T. Gigas*, the stimulation of hemocytes with LPS decreased the metabolites abundantly. Statistical analysis using Tukey shows that the changes are significant in *T. gigas* but not *C. rotundicauda.* In a study conducted by Song et al.^35^, monoglyceride and diglyceride were shown to have antiviral and antibacterial properties and act as emulsifiers to increase the digestibility of dietary lipids. Its supplementation could also effectively reduce fat loss, decrease inflammatory factor levels, and control total cholesterol concentrations during lactation^35^. In brown adipose tissue, L-Carnitine helps increase TG and diglyceride levels and reduces glycerophospholipids and sphingolipids.

L-Iditol, Fig. 5f increased after stimulation with LPS in both species. Statistical analysis using Tukey shows that only changes *in T. gigas* are significant. Studies regarding L-iditol alone are underreported. Only two relevant studies have been reported regarding L-Iditol, the angiosperm*- Yunnanopilia longistaminata*, a new plant source for L-iditol and taxanes^36^. Secondly, a series of quaternary diammonium salt derivatives of 1,4:3,6-dianhydro-l-iditol were synthesized, and two quaternary ammonium salts (QAS) with octyl and decyl residues exhibited antimicrobial activity^37^.

Octaprenyl diphosphate Fig. 5g also was found to be decreased after stimulation with LPS in hemocytes of both species and is statistically significant in the Tukey test. This metabolite is essential for the normal growth of *Escherichia coli *^38^. However, the relation between this metabolite and horseshoe crab is unknown.

Valine, Fig. 5h, was found to be increased in *C. rotundicauda;* meanwhile, in *T. gigas*, it was found to be decreased after stimulation with LPS. However, the changes were not statistically significant when a Tukey comparison was conducted. Despite that, this metabolite is abundant in *T. gigas,* which means it can be isolated in this species if needed. Valine has extensive industrial applications and is an intermediate for synthesizing agricultural pesticides and semisynthetic veterinary antibiotics^39^. *Bacillus cereus* could have a potential for industrial production of valine under optimized conditions^39^. Dietary L-Valine supplementation modulates the inflammatory response and microbial metabolites^40^.

Anthranilate Fig. 5i increased in *C. rotundicauda* and decreased in *T. gigas* after hemocyte stimulation with LPS. However, analysis using Tukey shows that the metabolite changes are not significant. Despite that, this metabolite was found to be abundant in *T. gigas.* This metabolite is widely used as a precursor in producing dyes, fragrances, plastics, and pharmaceutical compounds^41,42^. Microorganisms produce Anthranilate as an intermediate in the tryptophan biosynthetic pathway^41^. It has various biological activities, such as anti-inflammatory, antineoplastics, anti-malarial, and has α-glucosidase inhibitory properties^43^. Methyl anthranilate (2-aminobenzoic acid methyl ester) irritates birds' senses of taste and smell^44^, protecting sweet cherry orchards against birds. Anthranilate also increased the antibiotic susceptibility of other species of bacteria, such as *Escherichia coli*, *Salmonella enterica*, *Bacillus subtilis,* and *Staphylococcus aureus*^45^. Evaluating the antifungal activity in vitro of the active films containing methyl anthranilate showed great effectiveness against *Penicillium expansum* and *Botrytis cinerea*, demonstrating the potential applicability of the developed films for active food packaging. Evodileptin B (1) is a natural anthranilate derivative isolated from the ethanol extract of the aerial parts of *Evodia lepta* (Spreng.) Merr., a traditional medicinal plant of the family *Rutaceae*^46^. Evodileptin B has solid neuroprotective properties and may help treat Parkinson's Disease^46^. Linalyl anthranilate (LNA) generates reactive oxygen species, initiates lipid peroxidation, and damages the bacterial membrane, resulting in intracellular leakage and eventually killing *Klebsiella pneumoniae*^47^. Another study of Anthranilate is a novel anthranilate analogue (SI-W052) that inhibited LPS-induced tumour necrosis factor (TNF)-α and interleukin (IL)-6 on microglia^48^. However, further studies must be conducted to understand its relationship with horseshoes crab metabolites.

N-acetyllactosamine Fig. 5j was found to be decreased in *C. rotundicauda*, after being stimulated with LPS, but it increased in *T. gigas.* However, the Tukey test shows that the increment and reduction are not statistically significant. N-acetyllactosamine (LacNAc), specifically β-d-galactopyranosyl-1,4-N-acetyl-d-glucosamine, is a unique acyl-amino sugar and a critical structural unit in human milk oligosaccharides, an antigen component of many glycoproteins, and an active antiviral property for the development of effective drugs against viruses^49^. The 6-sulfo -N-acetyllactosamine was found to inhibit the binding of the SARS-CoV-2 spike protein S1 subunit with blood group A RBCs and reduce the interaction between the spike protein S1 subunit and Angiotensin-converting enzyme 2 (ACE2) in SARS-CoV-2 infection^50^.

We performed the t-test to analyze each species' pre- and post-LPS treatment metabolite changes. Only three metabolites exhibited a significant increase—specifically, the increments in L-iditol and Diacylglycerophospholipid in *T. gigas.* Meanwhile, dtMP deoxythymidylic acid was statistically significant in *C. rotundicauda* (Table S1).

Subsequent t-test analysis revealed that only three exhibited statistically significant decreases among eleven metabolites, showing a decrement pattern. These significant decrements were observed in 7,8-Dihydroxanthopterin, Phosphatidylinositol PI (18:3(6Z,9Z,12Z)/22:3(10Z,13Z,16Z)), and octaprenyl diphosphate in *T. gigas*, while in *C. rotundicauda*, the only metabolite with a significant decrement was octaprenyl diphosphate (Table S1).

Following LPS stimulation, a contrasting abundance pattern in 10 metabolites was observed in both species, with *C. rotundicauda* exhibiting an increase while *T. gigas* showed a decrease pattern. The metabolites were N-(1-Deoxy-1-fructosyl) tryptophan, 4-Hydroxy-5-(3″,5″-dihydroxyphenyl)-valeric acid-O-glucuronide, Contignasterol, Proline betaine (stachydrine), Diglyceride, Canavaninosuccinate, Stearoylglycerone phosphate, N-acetylactosamine, Valine, and Anthranilate. However, the t-test results indicated that only the change in Diglyceride in *T. gigas* was statistically significant (Table S1).

In another contrasting abundance pattern case, two metabolites, namely Phospholipid inositol PI (22:4(7Z,10Z,13Z,16Z)/16:1(9Z)) and Glycerophosphoinositol, exhibited a decrease in *C. rotundicauda* but an increase in *T. gigas* following LPS stimulation. However, t-test analysis indicated that none of these changes were statistically significant in either species (Table S1).

Another contrasting abundance pattern after LPS stimulation showed no changes in the metabolite's phospholipid inositol PI (21:0/20:2(11Z,14Z)) and PG (18:4(6Z,9Z,12Z,15Z)/14:1(9Z)) expression in C*. rotundicauda*, whereas *T. gigas* exhibited a decrease pattern in both metabolites. However, the t-test revealed that only the decreased metabolite phospholipid inositol PI (21:0/20:2(11Z,14Z)) in *T. gigas* showed a statistically significant. In the other cases, Mevaldate and Phosphatidylinositol PI(P-18:0V16:0) showed no change in expression in *C. rotundicauda* after LPS stimulation, but an increased pattern in *T. gigas* but none of these changes in *T. gigas* were significant (Table S1).

In summary, the significant changes observed in metabolites by the t-test, such as L-iditol, Diacylglycerophospholipid, and dtMP deoxythymidylic acid, following LPS stimulation in *T. gigas* and *C. rotundicauda*, suggest their involvement in crucial pathways; immune regulation, inflammation, and stress responses. These metabolites likely play roles in modulating immune cell function, membrane stability, or nucleotide metabolism, contributing to the immune response. The observed differences in metabolite abundance between the two species indicate unique strategies in response to the immune challenge posed by LPS. Species-specific responses, exemplified by *T. gigas*' significant decreases in 7,8-Dihydroxanthopterin, Phosphatidylinositol PI, and octaprenyl diphosphate, and *C. rotundicauda's* specific decrease in octaprenyl diphosphate, highlight distinct molecular adaptations which evolutionary history, environmental factors, and ecological niches might influence. These species-specific strategies reflect *C. rotundicauda* and *T. gigas'* adaptations to diverse environments and immune challenges.

### Pathway enrichment analysis

**Figure 6:**
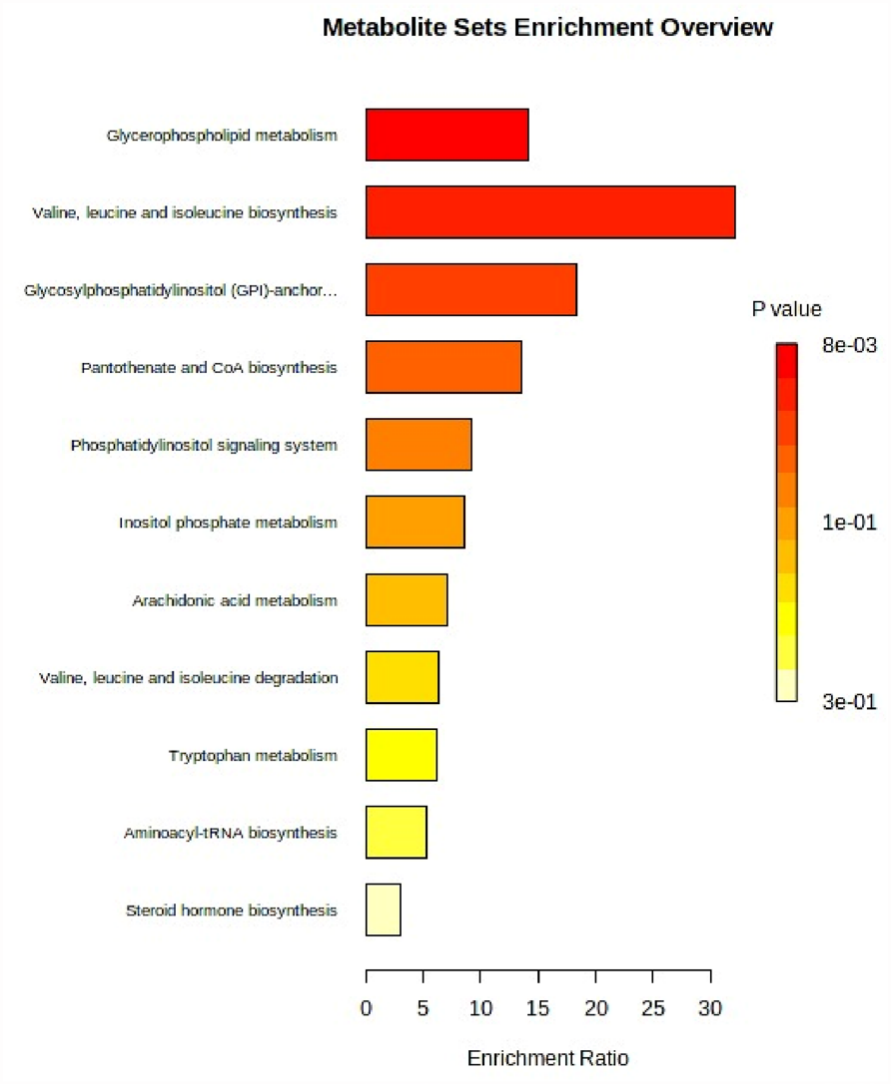
Overview of enrichment significant putative metabolites of horseshoe crabs.

MetaboAnalyst^51^ shows there are three significant pathways where the metabolites are found to be enriched, which are glycerophospholipid metabolism, valine, leucine, and isoleucine biosynthesis and glycosylphosphatidylinositol (GPI)-anchor biosynthesis (Fig. 6). Glycerophospholipids are the most abundant and dominant in cell membranes as they provide stability, fluidity, and permeability^52^. Moreover, they must function correctly as membrane proteins, receptors, and ion channels and as reservoirs for second messengers and their precursors. Thus, phosphatidylglycerol (12.46 min: 779.5176 *m/z*) in horseshoe crabs probably helps with cell membranes' excellent structure and signalling. On the other hand, the biosynthesis of valine, leucine, and isoleucine is crucial as they play critical roles in regulating energy homeostasis, nutrition metabolism, gut health, immunity, and disease in humans and animals^53^. Perhaps the existence of the metabolite valine (1.82 min: 140.0685 *m/z*) in horseshoe crabs also plays the same role as in other organisms. Glycosylphosphatidylinositol functions as an anchor to link cell membranes and proteins. These proteins act as enzymes, adhesion molecules, complement regulators, or co-receptors in signal transduction pathways^54^. The richness of this metabolite in this pathway is probably linked to the first hit, the glycerophospholipid metabolism, as it seems to work together. However, the metabolite name (1-Phosphatidyl-D-myo-inositol) that hit this pathway is not precisely the same as our putative metabolite Phosphatidylinositol (12.26 min: 911.5912 *m/z*). Despite that, as we refer to the KEGG database, it refers to the same metabolite but differs in name. The KEGG pathways involved are map00564 Glycerophospholipid metabolism, map00290 Valine, leucine and isoleucine biosynthesis and map00563 Glycosylphosphatidylinositol (GPI)-anchor biosynthesis^55–57^.

### Transcriptomics, proteomics, and metabolomics of hemocytes after LPS stimulation

Previous studies had conducted transcriptomics and proteomisc using hemocytes of horseshoe crabs^11,14^. The transcriptomics analysis reported 1338 genes were significantly upregulated, and 215 genes were downregulated after hemocytes were stimulated with LPS. Meanwhile, proteomics analysis reported 154 proteins were identified in the stimulated and non-stimulated form of hemocytes. From 154 proteins, 54 were found to be unique in hemocytes stimulated with LPS, and 25 were unique in non-stimulated form. Thirty- seven proteins are found to be shared in both conditions. Tachylectin-2, coagulogen, c-reactive proteins, histones, hemocyanin, and DNA polymerase, all of which play essential roles in the organism's innate immunity, were found to be differentially expressed in hemocytes after the LPS challenge^14^.

Gene ontology enrichment analysis from both studies showed several differentially expressed genes and proteins predictively involved in several metabolic processes such as cellular metabolic process, protein metabolic process, macromolecule metabolic process, and organonitrogen compound metabolic process. Indeed, our study showed several putative metabolites such as Androstenedione, 7,8-Dihydroneopterin, and Phosphatidylglycerol involved in the metabolic pathways by KEGG pathway analysis. Metabolites such as 7,8-Dihydroxanthopterin and Harman involved in the KEGG biosynthesis pathway, which the pathway was also enriched in a study by Sarmiento et al.^11^ at the gene level.

Nevertheless, it requires extensive study to understand the metabolites pathway and its functions to correlate our findings with previous findings at the gene and protein levels. Despite that, several metabolites such as Harman, L-Iditol, contignasterol, valine, Anthranilate, N-acetylactosamine, and Glycerophosphoinositol could be validate in further studies as they have immense benefits.

## Conclusions

Overall, this study successfully profiled the putative metabolites of horseshoe crab hemocytes stimulated with LPS and without stimulation with LPS. The result identified thirty-seven differentially abundant putative metabolites. Several putative metabolites, such as L-iditol, Diglyceride and Octaprenyl diphosphate, increased and decreased in abundance after LPS stimulation. Furthermore, metabolites such as Anthranilate and valine are more abundant in *T. gigas* than *C. rotundicauda*. Various medicinal values are reported from these metabolites. Examples of metabolites are Harman, L-Iditol, contignasterol, valine, Anthranilate, N-acetylactosamine, and Glycerophosphoinositol. Harman is a metabolite that has anti-cancer properties and bactericides. L-Iditol exhibited antimicrobial activity. Contignasterol acts as an anti-asthma agent. Anthranilate acts as an anti-inflammatory, antineoplastic, anti-malarial, and antifungal. Next, valine is an agricultural pesticide and a semisynthetic veterinary antibiotic. These findings emphasize a species-specific metabolic response, highlighting the complexity of host-specific reactions to LPS stimulation in these two species. Those metabolites merit further investigation for the validation study.

### Supplementary Information

Supplementary Table 1.

## Data Availability

The datasets used and/or analyzed during the current study available from the corresponding author on reasonable request.
