# Supplementary material for: Comparative putative metabolites profiling of Tachypleus gigas and Carcinoscorpius rotundicauda hemocytes stimulated with lipopolysaccharide
Source: Sci Rep. 2024 Feb 17;14:3968. doi: 10.1038/s41598-024-54279-3 (PMC10874427; doi:10.1038/s41598-024-54279-3)
Supplement: Supplementary file 1 — Supplementary Table 1. [file 41598_2024_54279_MOESM1_ESM.docx]

Table S1. Comparative analysis based on T-test between stimulated and non-stimulated with LPS of C. rotundicauda and T. gigas.

| No | The retention time of the  precursor ion and mass charge ratio (*m/z*) | Putatively identified  metabolites | Treatments | Mean  value | P -value  (<0.05) | Conclusion | Pattern of abundance |
| --- | --- | --- | --- | --- | --- | --- | --- |
| 1. | 6.16 min: 204.0492 | 7,8-Dihydroxanthopterin | *C. rotundicauda*  non-stimulated | 1032377 | 0.1909 | Not significant | Decrease |
|  |  |  | *C. rotundicauda*  stimulated | 854678 |  |  |  |
|  |  |  | *T- gigas*  non-stimulated | 444080 | 0.0061 | Significant |  |
|  |  |  | *T- gigas*  stimulated | 764739 |  |  |  |
| 2. | 2.28 min: 381.1506 | (N-(1-Deoxy-1-fructosyl) tryptophan) | *C. rotundicauda*  non-stimulated | 20197 | 0.3864 | Not significant | Increase |
|  |  |  | *C. rotundicauda*  stimulated | 150883 |  |  |  |
|  |  |  | *T- gigas*  non-stimulated | 127025 | 0.9554 | Not significant | Decrease |
|  |  |  | *T- gigas*  stimulated | 168087 |  |  |  |
| 3. | 1.83 min: 205.0693 | Harman | *C. rotundicauda*  non-stimulated | 581191 | 0.6012 | Not significant | Decrease |
|  |  |  | *C. rotundicauda*  stimulated | 521441 |  |  |  |
|  |  |  | *T- gigas*  non-stimulated | 292490 | 0.6038 | Not significant |  |
|  |  |  | *T- gigas*  stimulated | 352042 |  |  |  |
| 4. | 1.98 min: 403.1315 | 4-Hydroxy-5-(3'',5''-dihydroxyphenyl)-valeric acid-O-glucuronide | *C. rotundicauda*  non-stimulated | 36639 | 0.5099 | Not significant | Increase |
|  |  |  | *C. rotundicauda*  stimulated | 116200 |  |  |  |
|  |  |  | *T- gigas*  non-stimulated | 64964 | 0.9334 | Not significant | Decrease |
|  |  |  | *T- gigas*  stimulated | 97292 |  |  |  |
| 5. | 12.30 min: 867.5669 | Phosphatidylcholine,  PC(DiMe(11,5)/DiMe (9,3)) | *C. rotundicauda*  non-stimulated | 919125 | 0.9702 | Not significant | Decrease |
|  |  |  | *C. rotundicauda*  stimulated | 891534 |  |  |  |
|  |  |  | *T- gigas*  non-stimulated | 1036197 | 0.9892 | Not significant |  |
|  |  |  | *T- gigas*  stimulated | 1055582 |  |  |  |
| 6. | 12.26 min: 911.5912 | Phosphatidylinositol PI (18:3(6Z,9Z,12Z)/22:3(10Z,13Z,16Z)) | *C. rotundicauda*  non-stimulated | 946368 | 0.1778 | Not significant | Decrease |
|  |  |  | *C. rotundicauda*  stimulated | 900588 |  |  |  |
|  |  |  | *T- gigas*  non-stimulated | 924547 | 0.0296 | Significant |  |
|  |  |  | *T- gigas*  stimulated | 990103 |  |  |  |
| 7. | 12.39 min: 509.3332 | Contignasterol | *C. rotundicauda*  non-stimulated | 90789 | 0.5449 | Not significant | Increase |
|  |  |  | *C. rotundicauda*  stimulated | 163415 |  |  |  |
|  |  |  | *T- gigas*  non-stimulated | 154502 | 0.5575 | Not significant | Decrease |
|  |  |  | *T- gigas*  stimulated | 225994 |  |  |  |
| 8. | 12.46 min: 779.5176 | Phosphatidylglycerol PG (15:1(9Z) \/22:6(4Z,7Z,10Z,13Z,16Z19Z)) | *C. rotundicauda*  non-stimulated | 1111391 | 0.9366 | Not significant | Increase |
|  |  |  | *C. rotundicauda*  stimulated | 1123212 |  |  |  |
|  |  |  | *T- gigas*  non-stimulated | 1249177 | 0.7835 | Not significant |  |
|  |  |  | *T- gigas*  stimulated | 1230035 |  |  |  |
| 9. | 1.90 min: 287.1969 | Androstenedione | *C. rotundicauda*  non-stimulated | 70440 | 0.7409 | Not significant | Increase |
|  |  |  | *C. rotundicauda*  stimulated | 100311 |  |  |  |
|  |  |  | *T- gigas*  non-stimulated | 7354 | >0.9999 | Not significant |  |
|  |  |  | *T- gigas*  stimulated | 5975 |  |  |  |
| 10. | 8.29 min: 387.0849 | Methyl 18-bromo-15E,17E-octadecadien-5,7-diynoate | *C. rotundicauda*  non-stimulated | 298405 | 0.2285 | Not significant | Decrease |
|  |  |  | *C. rotundicauda*  stimulated | 216328 |  |  |  |
|  |  |  | *T- gigas*  non-stimulated | 133598 | 0.98084 | Not significant |  |
|  |  |  | *T- gigas*  stimulated | 149466 |  |  |  |
| 11. | 12.19 min: 999.6454 | Phosphatadylinositol PI (22:0\/22:1(11Z)) | *C. rotundicauda*  non-stimulated | 979849 | 0.9348 | Not significant | Decrease |
|  |  |  | *C. rotundicauda*  stimulated | 959552 |  |  |  |
|  |  |  | *T- gigas*  non-stimulated | 896534 | 0.0501 | Not significant |  |
|  |  |  | *T- gigas*  stimulated | 993220 |  |  |  |
| 12. | 12.37 min: 823.5371 | Phosphatadylinositol PI(P-18:0V16:0) | *C. rotundicauda*  non-stimulated | 1000870 | 0.9989 | Not significant | Decrease |
|  |  |  | *C. rotundicauda*  stimulated | 999290 |  |  |  |
|  |  |  | *T- gigas*  non-stimulated | 1111412 | 0.3016 | Not significant |  |
|  |  |  | *T- gigas*  stimulated | 1077485 |  |  |  |
| 13. | 1.93 min: 144.1021 | Proline betaine (stachydrine) | *C. rotundicauda*  non-stimulated | 54116 | 0.4613 | Not significant | Increase |
|  |  |  | *C. rotundicauda*  stimulated | 94716 |  |  |  |
|  |  |  | *T- gigas*  non-stimulated | 31214 | 0.9947 | Not significant | Decrease |
|  |  |  | *T- gigas*  stimulated | 37862 |  |  |  |
| 14. | 13.24 min: 227.1267 | 3,4-Methylenesebacic acid | *C. rotundicauda*  non-stimulated | 654779 | 0.2422 | Not significant | Decrease |
|  |  |  | *C. rotundicauda*  stimulated | 603158 |  |  |  |
|  |  |  | *T- gigas*  non-stimulated | 582961 | 0.0752 | Not significant |  |
|  |  |  | *T- gigas*  stimulated | 651877 |  |  |  |
| 15. | 13.22 min: 677.4942 | Diglyceride DG (18:2n6/0:0/20:4n6) | *C. rotundicauda*  non-stimulated | 133092 | 0.9819 | Not significant | Increase |
|  |  |  | *C. rotundicauda*  stimulated | 141032 |  |  |  |
|  |  |  | *T- gigas*  non-stimulated | 88814 | 0.0142 | Significant | Decrease |
|  |  |  | *T- gigas*  stimulated | 161180 |  |  |  |
| 16. | 12.39 min: 531.3464 | Nerolidol-3-O-α-L-rhamnopyranosyl-(1→6)-β-D-glucopyranoside | *C. rotundicauda*  non-stimulated | 166250 | 0.7529 | Not significant | Increase |
|  |  |  | *C. rotundicauda*  stimulated | 217305 |  |  |  |
|  |  |  | *T- gigas*  non-stimulated | 136219 | 0.9999 | Not significant |  |
|  |  |  | *T- gigas*  stimulated | 134051 |  |  |  |
| 17. | 12.22 min: 955.6180 | Phospholipid inositol  PI (21:0\/20:2(11Z,14Z)) | *C. rotundicauda*  non-stimulated | 899097 | >0.9999 | Not significant | No change |
|  |  |  | *C. rotundicauda*  stimulated | 898474 |  |  |  |
|  |  |  | *T- gigas*  non-stimulated | 858146 | 0.0233 | Significant | Decrease |
|  |  |  | *T- gigas*  stimulated | 925707 |  |  |  |
| 18. | 12.55 min: 885.5786 | Phospholipid inositol  PI (22:4(7Z,10Z,13Z,16Z) \/16:1(9Z)) | *C. rotundicauda*  non-stimulated | 95335 | 0.1212 | Not significant | Decrease |
|  |  |  | *C. rotundicauda*  stimulated | 43483 |  |  |  |
|  |  |  | *T- gigas*  non-stimulated | 86898 | 0.9676 | Not significant | Increase |
|  |  |  | *T- gigas*  stimulated | 76805 |  |  |  |
| 19. | 7.90 min: 364.0847 | 4-Methylthiobutyl-desulfoglucosinolate | *C. rotundicauda*  non-stimulated | 1545 | 0.9999 | Not significant | Increase |
|  |  |  | *C. rotundicauda*  stimulated | 3454 |  |  |  |
|  |  |  | *T- gigas*  non-stimulated | 104173 | 0.0995 | Not significant |  |
|  |  |  | *T- gigas*  stimulated | 21875 |  |  |  |
| 20. | 1.83 min: 387.1471 | N-acetylactosamine | *C. rotundicauda*  non-stimulated | 95333 | 0.1759 | Not significant | Increase |
|  |  |  | *C. rotundicauda*  stimulated | 56006 |  |  |  |
|  |  |  | *T- gigas*  non-stimulated | 50159 | 0.8588 | Not significant | Decrease |
|  |  |  | *T- gigas*  stimulated | 35642 |  |  |  |
| 21 | 13.25 min: 339.2501 | 5,6-DHET | *C. rotundicauda*  non-stimulated | 43924 | 0.0072 | Significant | Decrease |
|  |  |  | *C. rotundicauda*  stimulated | 13935 |  |  |  |
|  |  |  | *T- gigas*  non-stimulated | 22083 | 0.0243 | Significant |  |
|  |  |  | *T- gigas*  stimulated | 47582 |  |  |  |
| 22. | 1.87 min: 183.0870 | L-Iditol | *C. rotundicauda*  non-stimulated | 226473 | 0.9076 | Not significant | Increase |
|  |  |  | *C. rotundicauda*  stimulated | 235611 |  |  |  |
|  |  |  | *T- gigas*  non-stimulated | 242547 | 0.0248 | Significant |  |
|  |  |  | *T- gigas*  stimulated | 199744 |  |  |  |
| 23. | 12.45 min: 713.4391 | PG (18:4(6Z,9Z,12Z,15Z}) \/14:1(9Z)) | *C. rotundicauda*  non-stimulated | 210548 | 0.9992 | Not significant | No change |
|  |  |  | *C. rotundicauda*  stimulated | 214481 |  |  |  |
|  |  |  | *T- gigas*  non-stimulated | 196894 | 0.3576 | Not significant | Decrease |
|  |  |  | *T- gigas*  stimulated | 247371 |  |  |  |
| 24. | 2.33 min: 293.1001 | Canavaninosuccinate | *C. rotundicauda*  non-stimulated | 0 | 0.7319 | Not significant | Increase |
|  |  |  | *C. rotundicauda*  stimulated | 19120 |  |  |  |
|  |  |  | *T- gigas*  non-stimulated | 26386 | 0.8950 | Not significant | Decrease |
|  |  |  | *T- gigas*  stimulated | 39370 |  |  |  |
| 25. | 8.76 min: 943.5543 | Glycerophosphoinositolphosphate PIP (16:0/20:2(11Z,14Z)) | *C. rotundicauda*  non-stimulated | 131381 | 0.4929 | Not significant | Decrease |
|  |  |  | *C. rotundicauda*  stimulated | 105524 |  |  |  |
|  |  |  | *T- gigas*  non-stimulated | 40852 | 0.6248 | Not significant |  |
|  |  |  | *T- gigas*  stimulated | 62736 |  |  |  |
| 26. | 11.52 min: 437.2495 | Stearoylglycerone phosphate | *C. rotundicauda*  non-stimulated | 39413 | 0.2035 | Not significant | Increase |
|  |  |  | *C. rotundicauda*  stimulated | 71647 |  |  |  |
|  |  |  | *T- gigas*  non-stimulated | 56063 | 0.9965 | Not significant | Decrease |
|  |  |  | *T- gigas*  stimulated | 59406 |  |  |  |
| 27. | 12.51 min: 929.6049 | Glycerophosphoinositol PI (19:0\/22:4  (7Z,10Z,13Z,16Z)) | *C. rotundicauda*  non-stimulated | 154344 | 0.7241 | Not significant | Decrease |
|  |  |  | *C. rotundicauda*  stimulated | 119742 |  |  |  |
|  |  |  | *T- gigas*  non-stimulated | 142899 | 0.9528 | Not significant | Increase |
|  |  |  | *T- gigas*  stimulated | 125663 |  |  |  |
| 28. | 11.23 min: 745.4335 | Octaprenyl diphosphate | *C. rotundicauda*  non-stimulated | 34176 | 0.0022 | Significant | Decrease |
|  |  |  | *C. rotundicauda*  stimulated | 12949 |  |  |  |
|  |  |  | *T- gigas*  non-stimulated | 13864 | 0.0488 | Significant |  |
|  |  |  | *T- gigas*  stimulated | 27987 |  |  |  |
| 29. | 13.38 min: 373.2712 | Cervonoyl ethanolamide | *C. rotundicauda*  non-stimulated | 175247 | 0.6667 | Not significant | Decrease |
|  |  |  | *C. rotundicauda*  stimulated | 154148 |  |  |  |
|  |  |  | *T- gigas*  non-stimulated | 140794 | 0.1812 | Not significant |  |
|  |  |  | *T- gigas*  stimulated | 179832 |  |  |  |
| 30. | 1.88 min: 147.0650 | Mevaldate | *C. rotundicauda*  non-stimulated | 40442 | 0.9999 | Not Significant | No change |
|  |  |  | *C. rotundicauda*  stimulated | 40500 |  |  |  |
|  |  |  | *T- gigas*  non-stimulated | 38707 | 0.0789 | Not Significant | Decrease |
|  |  |  | *T- gigas*  stimulated | 17736 |  |  |  |
| 31. | 1.82 min: 140.0685 | Valine | *C. rotundicauda*  non-stimulated | 9914 | 0.4822 | Not Significant | Increase |
|  |  |  | *C. rotundicauda*  stimulated | 22908 |  |  |  |
|  |  |  | *T- gigas*  non-stimulated | 36518 | 0.9968 | Not Significant | Decrease |
|  |  |  | *T- gigas*  stimulated | 38537 |  |  |  |
| 32. | 1.93 min: 309.1772 | Fructoselysine | *C. rotundicauda*  non-stimulated | 9314 | 0.3738 | Not Significant | Increase |
|  |  |  | *C. rotundicauda*  stimulated | 20595 |  |  |  |
|  |  |  | *T- gigas*  non-stimulated | 5483 | 0.8522 | Not Significant |  |
|  |  |  | *T- gigas*  stimulated | 0 |  |  |  |
| 33. | 1.96 min: 138.0558 | Anthranilate | *C. rotundicauda*  non-stimulated | 1455 | 0.9994 | Not Significant | Increase |
|  |  |  | *C. rotundicauda*  stimulated | 2141 |  |  |  |
|  |  |  | *T- gigas*  non-stimulated | 18169 | 0.9970 | Not Significant | Decrease |
|  |  |  | *T- gigas*  stimulated | 19331 |  |  |  |
| 34. | 14.12 min: 807.5674 | PA (22:1(11Z) \/22:4(7Z,10Z,13Z,16Z))-Diacylglycerophospholipid | *C. rotundicauda*  non-stimulated | 107870 | 0.0544 | Not Significant | Increase |
|  |  |  | *C. rotundicauda*  stimulated | 131688 |  |  |  |
|  |  |  | *T- gigas*  non-stimulated | 165370 | 0.0174 | Significant |  |
|  |  |  | *T- gigas*  stimulated | 136898 |  |  |  |
| 35. | 11.41 min: 359.0756 | 7,8-Dihydroneopterin 2'',3''-cyclic phosphate | *C. rotundicauda*  non-stimulated | 5937 | 0.07750 | Not Significant | Increase |
|  |  |  | *C. rotundicauda*  stimulated | 16916 |  |  |  |
|  |  |  | *T- gigas*  non-stimulated | 45583 | 0.0614 | Not Significant |  |
|  |  |  | *T- gigas*  stimulated | 14627 |  |  |  |
| 36. | 9.88 min: 313.0707 | (Indole-3-acetyl) aspartic acid | *C. rotundicauda*  non-stimulated | 11609 | 0.5030 | Not Significant | Increase |
|  |  |  | *C. rotundicauda*  stimulated | 31907 |  |  |  |
|  |  |  | *T- gigas*  non-stimulated | 45704 | 0.3209 | Not Significant |  |
|  |  |  | *T- gigas*  stimulated | 20520 |  |  |  |
| 37. | 2.45 min: 345.0350 | dtMP Deoxythymidylic acid | *C. rotundicauda*  non-stimulated | 4694 | 0.7321 | Not Significant | Increase |
|  |  |  | *C. rotundicauda*  stimulated | 11159 |  |  |  |
|  |  |  | *T- gigas*  non-stimulated | 34107 | 0.0332 | Significant |  |
|  |  |  | *T- gigas*  stimulated | 15371 |  |  |  |
